# Supplementary material for: Anthropogenic sulfate aerosol pollution in South and East Asia induces increased summer precipitation over arid Central Asia
Source: Commun Earth Environ. 2022 Dec 27;3(1):328. doi: 10.1038/s43247-022-00660-x (PMC9792934; doi:10.1038/s43247-022-00660-x)
Supplement: Supplementary file 1 — Supplementary Information [file 43247_2022_660_MOESM1_ESM.pdf]

Supplementary information

Anthropogenic sulfate aerosol pollution in South and East Asia induces increased summer precipitation over arid Central Asia

Xiaoning Xie[1,2], Gunnar Myhre[3], Drew Shindell[4], Gregory Faluvegi[5,6], Toshihiko Takemura[7], Apostolos Voulgarakis[8,9], Zhengguo Shi[1], Xinzhou Li[1], Xiaoxun Xie[1], Heng Liu[1,10], Xiaodong Liu[1,11], Yangang Liu[12]

1, SKLLQG, Institute of Earth Environment, Chinese Academy of Sciences, Xi'an, China

2, CAS Center for Excellence in Quaternary Science and Global Change, Xi'an, China

3, Center for International Climate and Environmental Research, Oslo, Norway

4, Nicholas School of the Environment, Duke University, Durham, USA

5, Center for Climate System Research, Columbia University, New York, NY, USA

6, NASA Goddard Institute for Space Studies, New York, NY, USA

7, Climate Change Science Section, Kyushu University, Fukuoka, Japan

8， Department of Physics, Imperial College London, South Kensington Campus, London, UK

9，School of Environmental Engineering, Technical University of Crete, Chania, Crete, Greece

10, Xi'an Institute for Innovative Earth Environment Research, Xi'an, China

11, College of Earth and Planetary Sciences, University of Chinese Academy of Sciences, Beijing, China

12, Environmental and Climate Sciences Department, Brookhaven National Laboratory, Upton, NY, USA

Correspondence: Xiaoning Xie (xnxie@ieecas.cn) and Yangang Liu (lyg@bnl.gov)

Supplementary Table 1 Asian sulfate-induced changes in precipitation and extreme precipitation over arid Central Asia (ACA). Percentage changes (%) induced by increasing Asian sulfate (SULx10Asia) in JJA total precipitation (PRECT), convective precipitation (PRECC), large-scale precipitation (PRECL), the extreme precipitation (R95p), the frequency and intensity contribution to R95p for individual models and multi-model mean (MMM) over the ACA region.

	PRECT	PRECC	PRECL	R95p	Frequency	Intensity
GISS-E2	2.0	0.5	3.6	-10.9	-12.5	-1.8
HadGEM3-GA4	18.9	15.9	36.9	16.6	19.4	1.5
IPSL-CM5A	11.8	12.6	11.6	4.1	4.0	1.8
MIROC	15.7	25.2	9.4	14.3	9.0	4.3
CAM4	19.5	24.8	9.7	12.5	20.5	-5.3
CAM5	16.2	14.2	19.9	14.2	14.1	-0.5
NorESM1	15.8	22.5	4.1	0.9	9.5	-4.2
MMM	14.3±6.0	16.5±8.7	13.6±11.6	7.4±10.0	9.2±11.2	-0.6±3.4

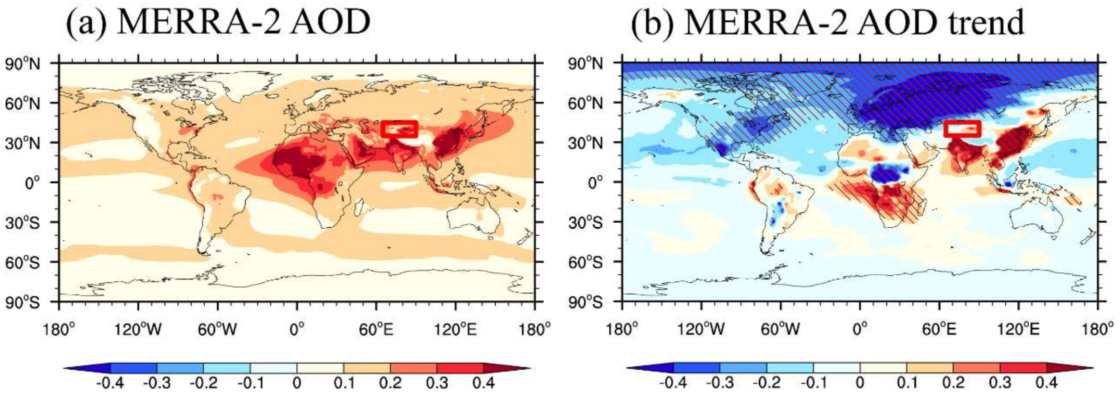

Supplementary Fig. 1 Climatology and trend of Aerosol optical depth (AOD). (a) Spatial pattern of annual mean AOD from 1980 to 2005 and **(b)** for annual AOD trend (per 100 years) during the same period based on MERRA-2, available through https://disc.gsfc.nasa.gov/datasets?project=MERRA-2. Red box in **(a)** and **(b)** indicates the ACA region and slanted lines in **(b)** represent significance at the 95% confidence level by a standard t-test.

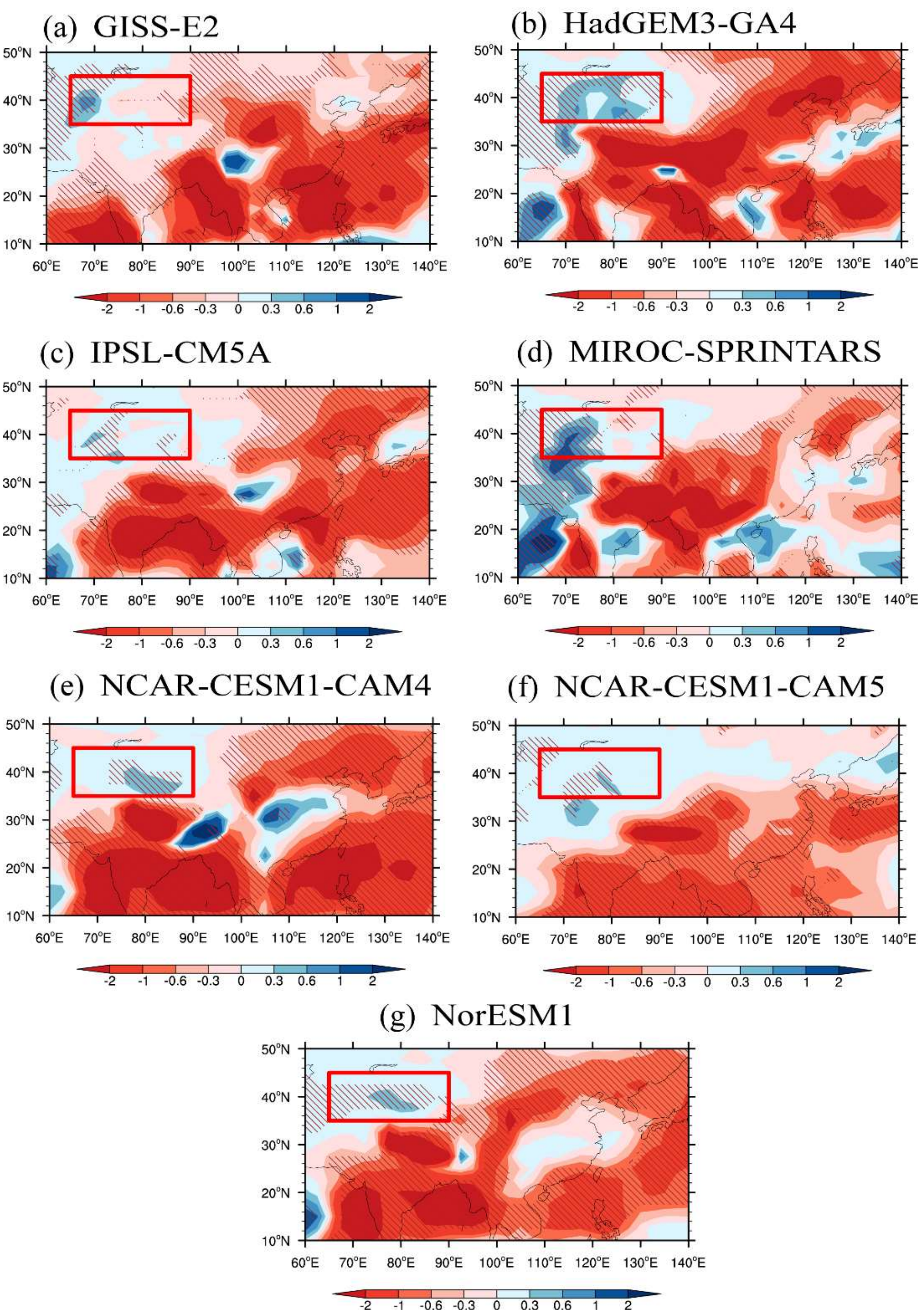

Supplementary Fig. 2 Asian sulfate-induced changes in precipitation over arid Central Asia (ACA) for individual models. Geographical distribution of changes induced by increasing Asian sulfate (SULx10Asia) in JJA total precipitation (mm day^{-1}) for **(a)** GISS-E2, **(b)** HadGEM3-GA4, **(c)** IPSL-CM5A, **(d)** MIROC-SPRINTARS, **(e)** NCAR-CESM1-CAM4, **(f)** NCAR-CESM1-CAM5, and **(g)** NorESM1. Red box in **(a)-(g)** indicates the ACA region and slanted lines represent significance at the 95% confidence level by a standard t-test.

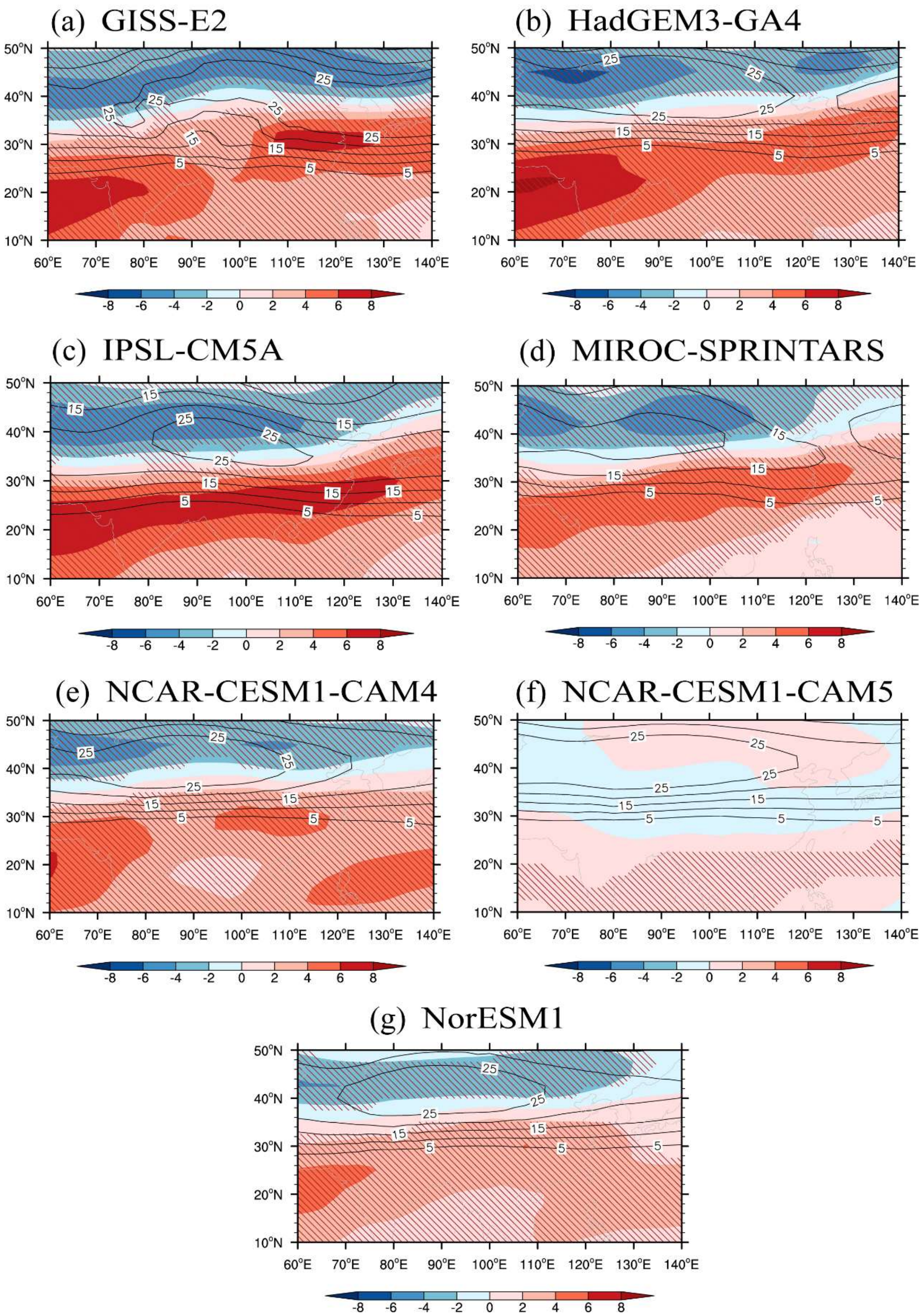

Supplementary Fig. 3 Asian sulfate-induced changes in westerlies for individual models. Same as Supplementary Fig. 2 but for JJA westerlies at 200 hPa (m s^{-1}). Thin black lines in **(a)-(g)** show the climatological westerlies and slanted lines represent significance at the 95% confidence level by a standard t-test.

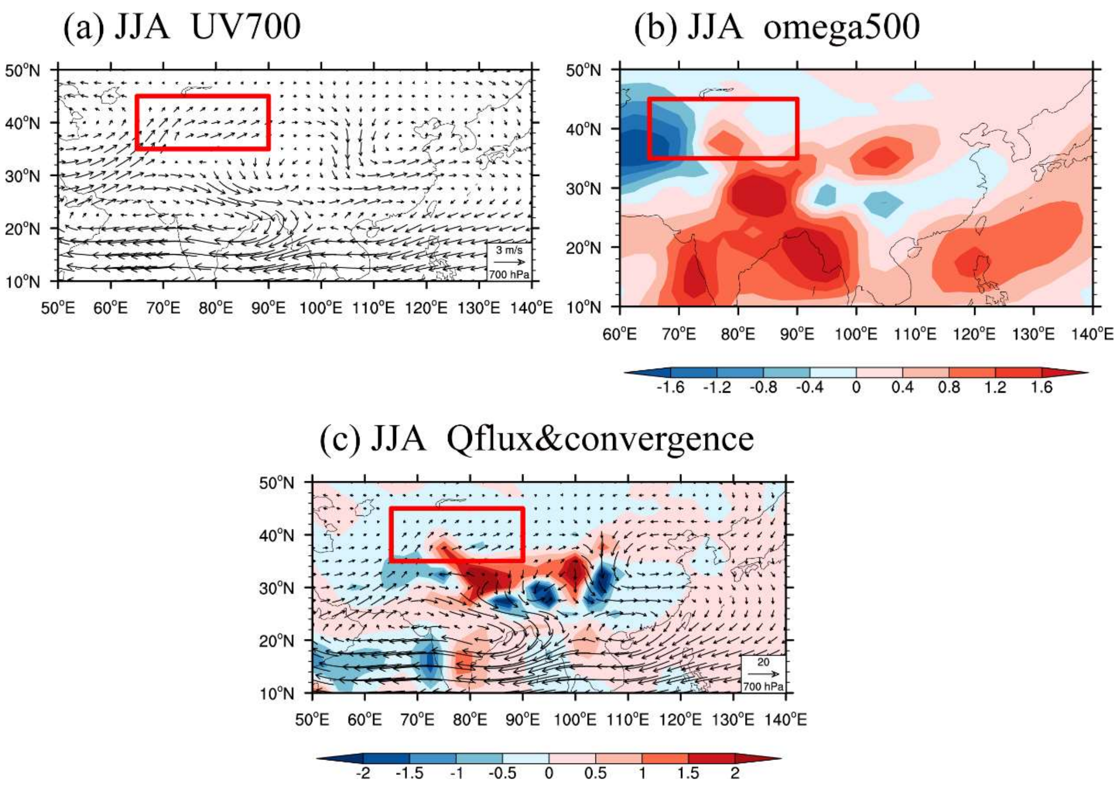

Supplementary Fig. 4 Asian sulfate-induced changes in wind field and water vapor flux. Geographical distribution of changes induced by increasing Asian sulfate (SULx10Asia) in **(a)** JJA 700 hPa wind field (UV700, m s^{-1}), **(b)** vertical velocity at 500 hPa (Omega500, 0.01 x Pa s^{-1}), and **(c)** water vapor flux at 700 hPa (vector, g (hPa m s)$^{-1}$) and water flux convergence at 700 hPa (shaded, 10^{-5} g (hPa m^2 s)$^{-1}$). Red box in **(a)**-**(c)** indicates the ACA region.

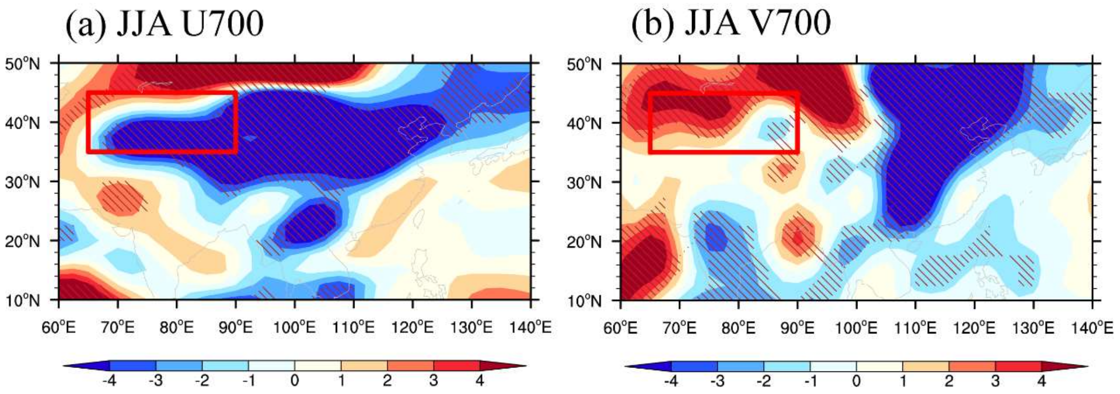

Supplementary Fig. 5 Observed trend of wind fields. Spatial pattern in trends of **(a)** JJA westerly winds and **(b)** JJA southerly winds at 700 hPa (m s^{-1} per 100 years) from 1961 to 2005 (NCEP/NCAR Reanalysis 1). Slanted lines in **(a)** and **(b)** represent significance at the 95% confidence level. Red box in **(a)** and **(b)** indicates the ACA region.

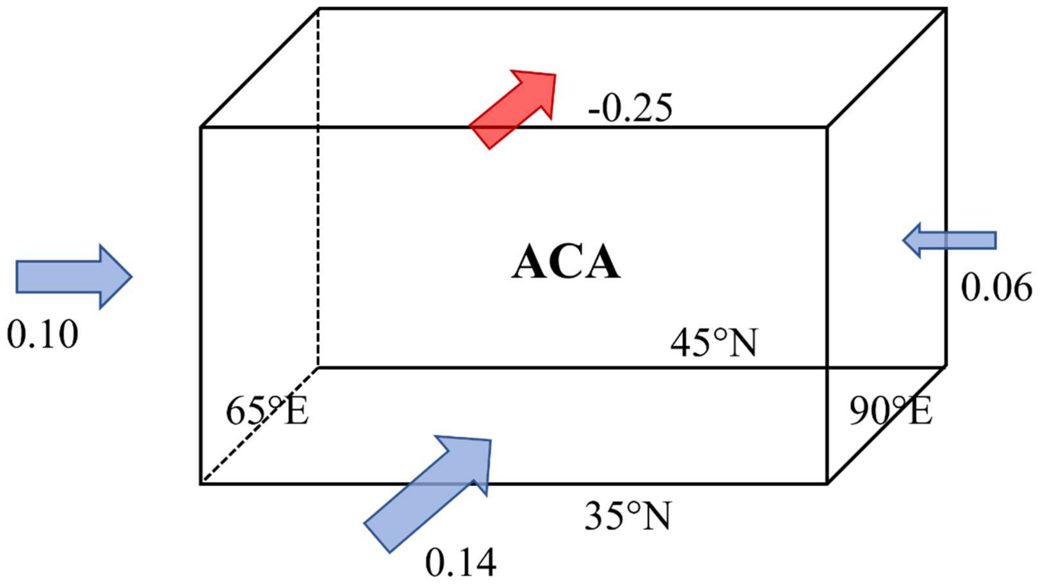

Supplementary Fig. 6 Asian sulfate-induced changes in water vapor fluxes. The changes induced by increasing Asian sulfate (SULx10Asia) of the multi-model mean (MMM) in JJA water vapor fluxes (mm day^{-1}), integrated between the surface and 300 hPa, through four boundaries of the ACA region. The ACA region is indicated with a box. The sign of the moisture budget changes is positive (negative) if the water vapor transports into (out of) the ACA box.

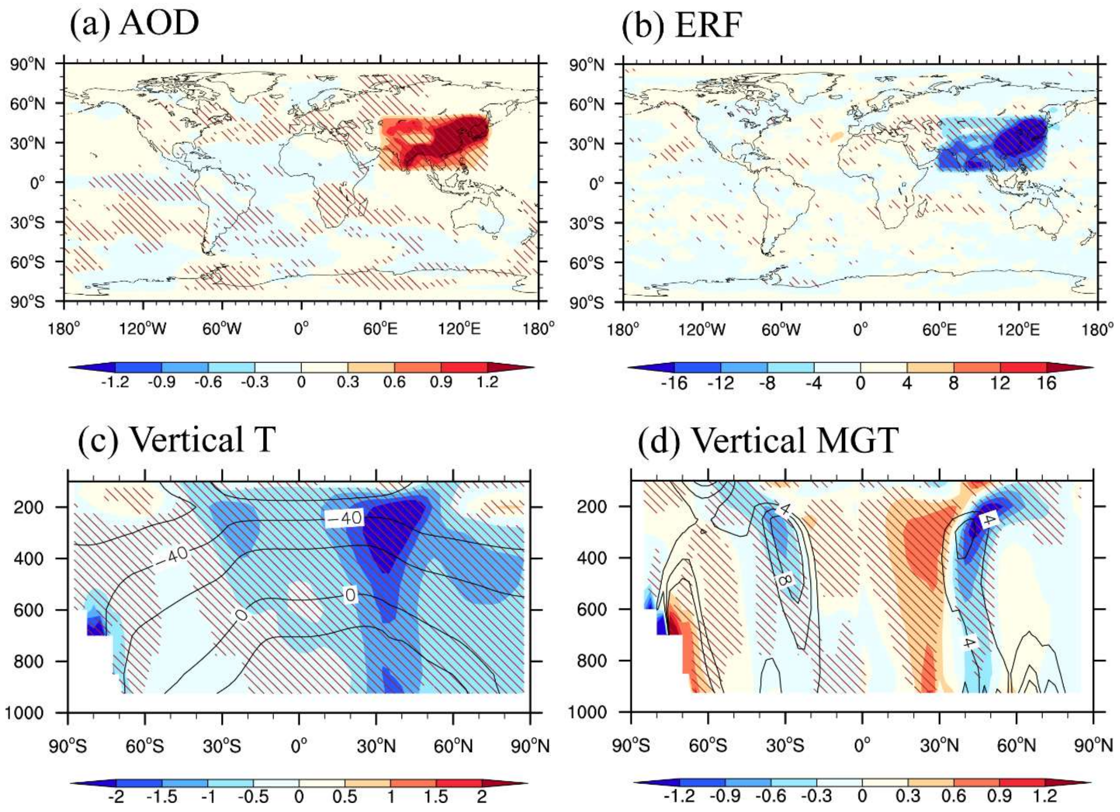

Supplementary Fig. 7 Asian sulfate-induced changes in radiative forcing and air temperature. The changes induced by increasing Asian sulfate (SULx10Asia) of the multi-model mean (MMM) in **(a)** JJA aerosol optical depth (AOD), **(b)** effective radiative forcing (ERF, W m^{-2}), **(c)** zonally mean temperature (T, ^{o}C), and **(d)** zonal mean temperature gradient (MGT, 10^{-3} ^{o}C km^{-1}). Thin black lines show the climatological temperature in **(c)** and temperature gradient in **(d)**. Slanted lines in **(a)-(d)** represent where MMM is more than 1 standard deviation of the PDRMIP models away from 0.

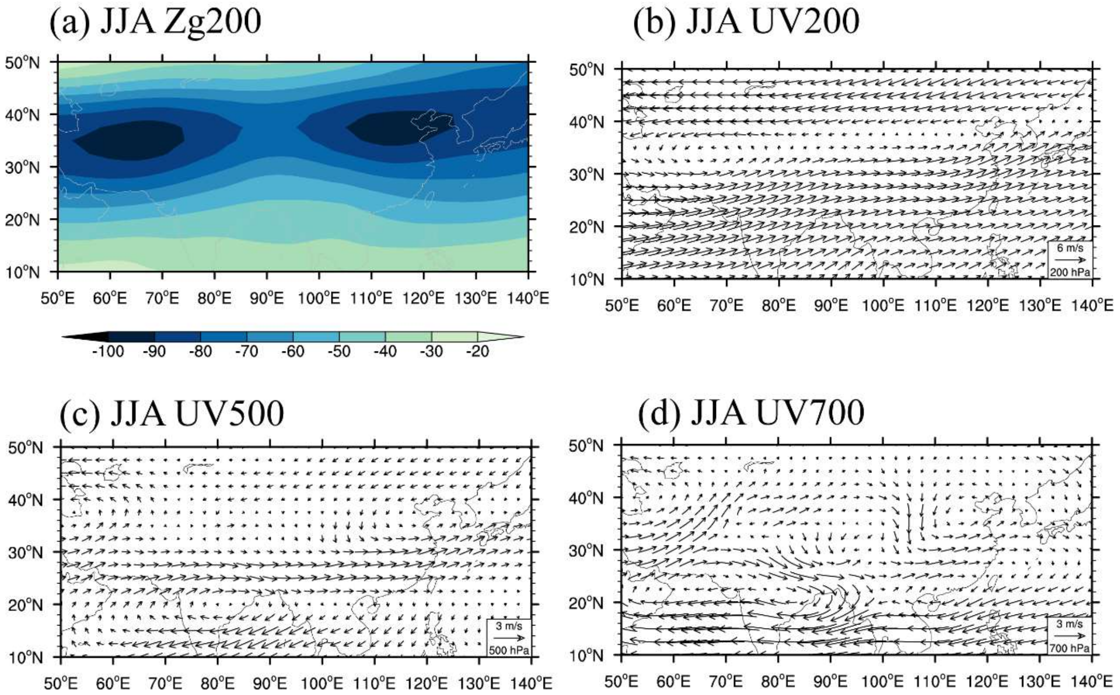

Supplementary Fig. 8 Asian sulfate-induced changes in geopotential height and wind field. The changes induced by increasing Asian sulfate (SULx10Asia) of the multi-model mean (MMM) in **(a)** JJA 200 hPa geopotential height (Zg200, gpm), **(b)** JJA 200 hPa wind field (UV200, m s^{-1}), **(c)** JJA 500 hPa wind field (UV500, m s^{-1}), and **(d)** JJA 700 hPa wind field (UV700, m s^{-1}).

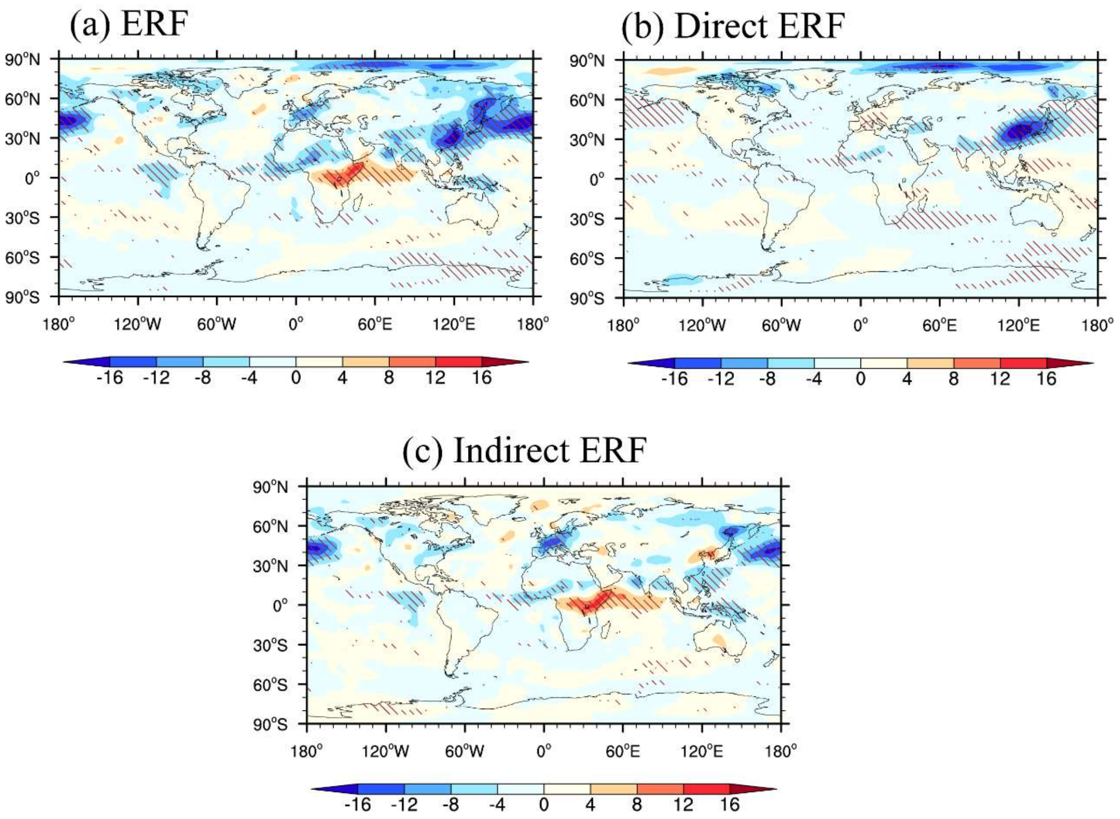

Supplementary Fig. 9 Asian sulfate-induced changes in aerosol radiative forcing. The changes induced by increasing Asian sulfate (SULx10Asia) in **(a)** total effective radiative forcing (ERF, W m^{-2}), **(b)** direct effective radiative forcing, and **(c)** indirect effective radiative forcing in NCAR-CESM1-CAM5, where total ERF = Direct ERF + Indirect ERF. Slanted lines in **(a)-(c)** represent significance at the 95% confidence level by a standard t-test.

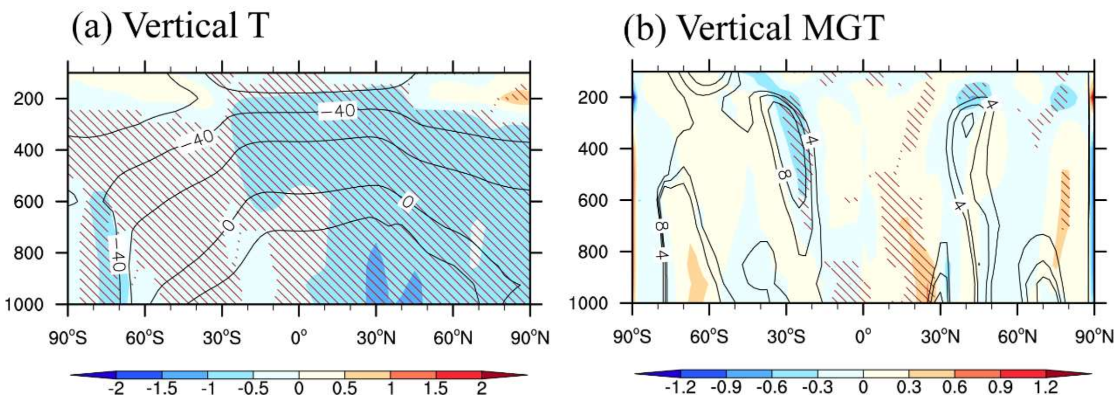

Supplementary Fig. 10 Asian sulfate-induced changes in air temperature. The changes induced by increasing Asian sulfate (SULx10Asia) of **(a)** JJA zonally mean temperature (T, °C) and **(b)** zonal mean temperature gradient (MGT, 10^{-3} °C km^{-1}) in NCAR-CESM1-CAM5. Thin black lines show the climatological temperature in **(a)** and temperature gradient in **(b)**. Slanted lines in **(a)** and **(b)** represent significance at the 95% confidence level by a standard t-test.

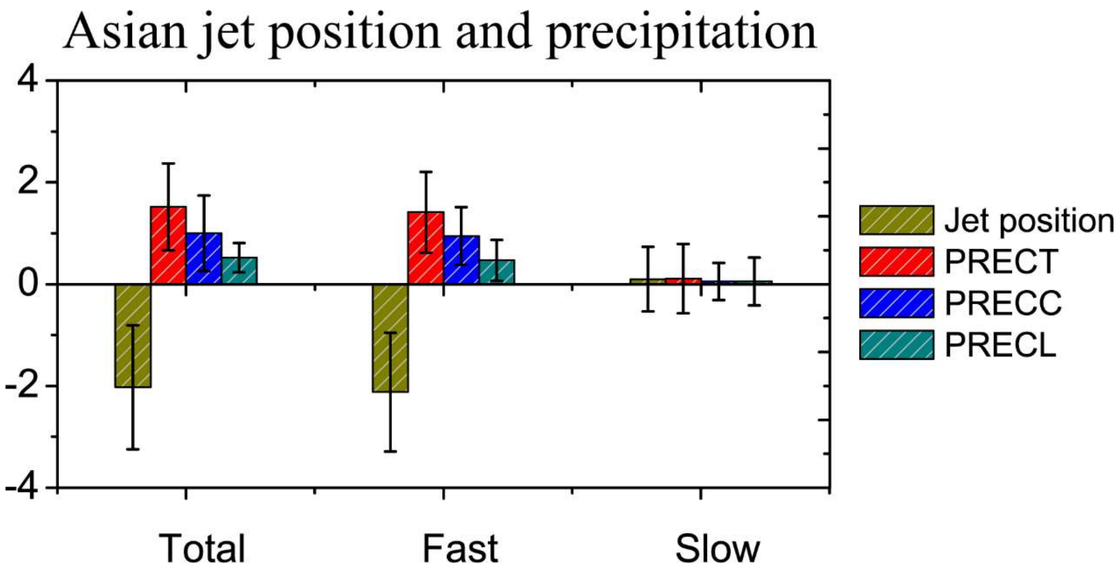

Supplementary Fig. 11 Asian sulfate-induced changes in Asian westerly jet position and precipitation for total, fast, and slow responses. Changes in JJA Asian westerly jet position (degree), JJA total precipitation (PRECT, 10^{-1} mm day^{-1}), convective precipitation (PRECC, 10^{-1} mm day^{-1}), and large-scale precipitation (PRECL, 10^{-1} mm day^{-1}) for the multi-model mean (MMM) over ACA. Error bars of MMM represent the standard deviation among the PDRMIP models.

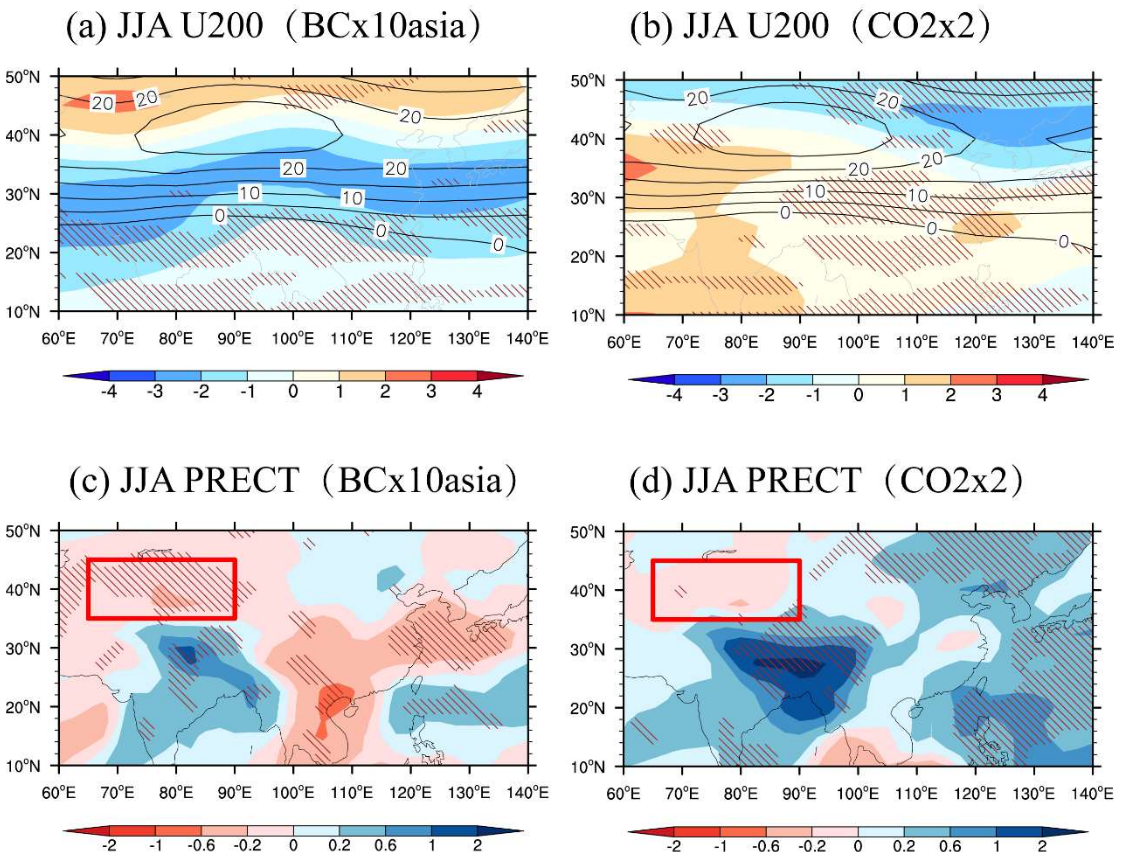

Supplementary Fig. 12 Changes in westerlies and precipitation induced by Asian black carbon and greenhouse gases. Asian black carbon-induced changes (BCx10asia) of the multi-model mean (MMM) in **(a)** JJA westerlies at 200 hPa (U200, m s^{-1}) and **(c)** JJA precipitation (mm day^{-1}). (b, d) But for GHGs (CO2x2). Thin black lines in **(a, b)** show the climatological westerlies and slanted lines in **(a)-(d)** represent where MMM is more than 1 standard deviation of the PDRMIP models away from 0. Red box in **(c)** and **(d)** indicates the ACA region.

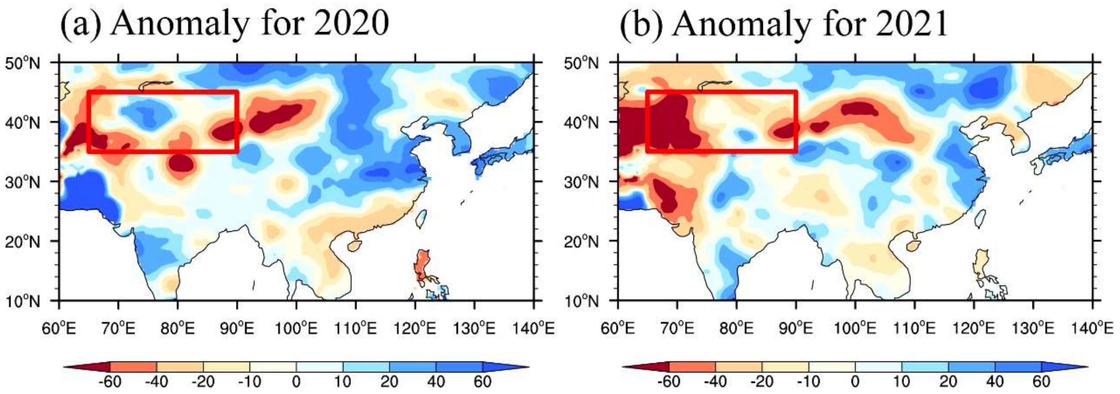

Supplementary Fig. 13 Observed JJA precipitation anomalies (%) for the years 2020 (a) and 2021 (b). The anomaly of precipitation is calculated by removing the climatology for the period 1979–2021 based on CRU TS Version 4.06. Red box in **(a)** and **(b)** indicates the ACA region.
